# Silent Barriers: The Hidden Cost of Discrimination in Black Men’s Health Care Utilization

**DOI:** 10.1093/geroni/igaf122.1687

**Published:** 2025-12-31

**Authors:** Ryon Cobb

**Affiliations:** Rutgers University, New Brunswick, New Jersey, United States

## Abstract

**Background:**

Everyday discrimination is a well-documented risk factor for poor health outcomes, yet its impact on healthcare utilization among older Black men remains understudied. While discriminatory experiences shape healthcare behaviors, little is known about how distinct patterns of discrimination influence hospital visits. This study employs latent class analysis to identify discrimination subgroups and examines their association with healthcare utilization, addressing a critical gap in minority health research.

**Methods:**

Using data from the 2010/2012 Health and Retirement Study, I conducted a latent class analysis to identify discrimination patterns among Black men aged 50 and older (N = 843). Negative binomial regression models assessed the association between discrimination classes and hospital visits, adjusting for sociodemographic and health factors.

**Results:**

The mean number of hospital visits was slightly under 13. Three distinct discrimination classes emerged: Low Discrimination (35.85%), Race/Ancestry Discrimination (32.4%), and Broad Discrimination (31.75%). Compared to men in the Low Discrimination class, those in the Race/Ancestry Discrimination group had significantly lower odds of hospital visits. No significant differences were observed between the Broad Discrimination and Low Discrimination groups.

**Implications:**

Findings highlight the need for healthcare interventions that address multiple forms of discrimination. Providers should implement culturally responsive practices and integrate discrimination screening into clinical care. Future research should investigate the mechanisms through which discrimination shapes healthcare-seeking behaviors, informing targeted interventions to reduce disparities and improve health outcomes for older Black men.

